# Assessment of neurotransmitter release in human iPSC-derived neuronal/glial cells: a missing in vitro assay for regulatory developmental neurotoxicity testing

**DOI:** 10.1016/j.reprotox.2023.108358

**Published:** 2023-04

**Authors:** Chiara Cervetto, Francesca Pistollato, Sarah Amato, Emilio Mendoza-de Gyves, Anna Bal-Price, Guido Maura, Manuela Marcoli

**Affiliations:** a Department of Pharmacy (DIFAR), Section of Pharmacology and Toxicology, University of Genoa, Italy; b Interuniversity Center for the Promotion of the 3Rs Principles in Teaching and Research, Centro 3R, Pisa, Italy; c European Commission, Joint Research Centre, JRC, Ispra, Italy

**Keywords:** Neurotransmitters, Glutamate, Vesicular release, Aspartic acid, Multi-electrode array, Human neural stem cells, Neurons, Glia, Developmental neurotoxicity

## Abstract

Human induced pluripotent stem cell (hiPSC)-derived neural stem cells (NSCs) and their differentiated neuronal/glial derivatives have been recently considered suitable to assess in vitro developmental neurotoxicity (DNT) triggered by exposure to environmental chemicals. The use of human-relevant test systems combined with in vitro assays specific for different neurodevelopmental events, enables a mechanistic understanding of the possible impact of environmental chemicals on the developing brain, avoiding extrapolation uncertainties associated with in vivo studies. Currently proposed in vitro battery for regulatory DNT testing accounts for several assays suitable to study key neurodevelopmental processes, including NSC proliferation and apoptosis, differentiation into neurons and glia, neuronal migration, synaptogenesis, and neuronal network formation. However, assays suitable to measure interference of compounds with neurotransmitter release or clearance are at present not included, which represents a clear gap of the biological applicability domain of such a testing battery. Here we applied a HPLC-based methodology to measure the release of neurotransmitters in a previously characterized hiPSC-derived NSC model undergoing differentiation towards neurons and glia. Glutamate release was assessed in control cultures and upon depolarization, as well as in cultures repeatedly exposed to some known neurotoxicants (BDE47 and lead) and chemical mixtures. Obtained data indicate that these cells have the ability to release glutamate in a vesicular manner, and that both glutamate clearance and vesicular release concur in the maintenance of extracellular glutamate levels. In conclusion, analysis of neurotransmitter release is a sensitive readout that should be included in the envisioned battery of in vitro assays for DNT testing.

## Highlights

• Human iPSC-derived NSCs and their neuronal/glial derivatives are suitable for DNT testing.
• HPLC-based assay can be used to measure neurotransmitter release in human NSCs.
• Differentiated NSCs release glutamate through a synaptic vesicle mechanism.
• BDE47, lead and chemical mixtures differentially perturb glutamate release and clearance in differentiated NSCs.
• Analyses of neurotransmitters should be included in the battery of assays for regulatory DNT.

## 1 Introduction

Human induced pluripotent stem cell (hiPSC)-derived neural stem cells (NSCs) and their differentiated neuronal/glial derivatives have been proven useful biological test systems to assess developmental neurotoxicity (DNT) using an in vitro approach [1], [2], [3], [4], [5], [6]. The use of human relevant test systems combined with assays specific for different stages of brain development enables a mechanistic understanding of the possible impact of environmental chemicals on key neurodevelopmental processes, which cannot be assessed using traditional in vivo test guidelines (e.g., OECD TG 426 [7] or OECD TG 443 [8]), due to the lack of mechanistic information and extrapolation uncertainties associated with interspecies differences.

Rather than a one-to-one replacement of in vivo models with human-based in vitro systems, it is believed that a combination (or a battery) of different assays based on human NSC-derived mixed neuronal/glial models could be suitable to mimic to a certain extent the biological complexity of the developing brain. These in vitro models permit to investigate the impact of chemicals on key processes of human brain development [9], [10], [11], [12], such as NSC commitment, proliferation, migration, differentiation towards neurons and glia, neurite outgrowth and synaptogenesis, neuronal network formation and function and, as reported in this study, the release of neurotransmitters. These processes are modulated by several neurodevelopmental signalling pathways, such as Notch, BMP, Wnt, Sonic hedgehog and ERK-CREB-BDNF [3], [4].

**Fig. 1:**
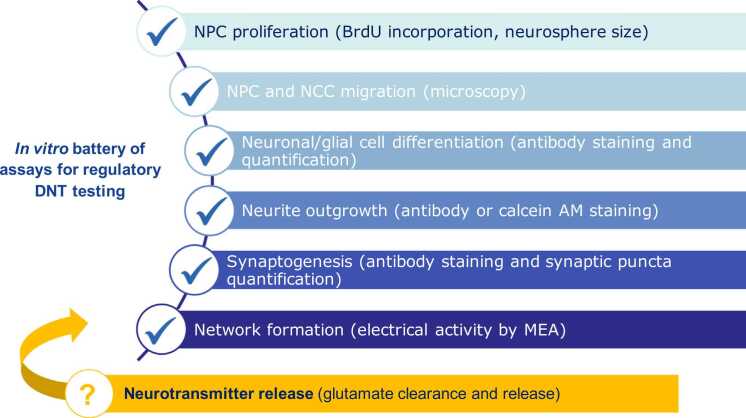
Current battery of in vitro assays included in the draft of the OECD Guidance Document considered suitable for regulatory DNT testing. Neural progenitor cells (NPC) and neural crest cells (NCC) proliferation, migration, neuronal/glial differentiation, neurite outgrowth and synaptogenesis assessed by different readouts (e.g., antibody staining, microscopy and quantification analyses), as well as network formation measured by multi-electrode array (MEA), are considered as ready-to-use assays for different regulatory purposes [14]. Measurement of neurotransmitter release (by HPLC) investigated in the present study (yellow box), represents a sensitive and complementary functional endpoint suitable to study DNT effects.

In the recently published EFSA document focused on the “*Establishment of an* a priori *protocol for an in vitro testing battery*”, several DNT assays are described, i.e., neural crest cell (NCC) migration, neural progenitor cell (NPC) proliferation and apoptosis, radial glia migration, NPC differentiation into neurons, neuronal migration and neuronal morphology, synaptogenesis, oligodendrocyte differentiation and migration, and neuronal network formation with glutamatergic and GABA-ergic functions. These assays served as a base for drafting the OECD Guidance document on the DNT in vitro battery (IVB) of assays, including their application and data interpretation, which will be shortly finalized [9], [13], [14], [15], [16], [17], [18], [19] However, assays suitable to measure interference of compounds with neurotransmitters, growth factors, neurotrophins, or ion channels/receptors are not included in the current DNT IVB, which represents a clear gap of the biological applicability domain of such testing battery (Fig. 1).

Neurotransmitters play a fundamental role as synaptic chemical messengers in the mature brain modulating neuronal network function, and during brain development [20], [21], [22], [23], controlling cell proliferation [24], neuronal migration [25], [26], neuronal differentiation [27], neurite outgrowth [28], synaptic maturation [29], and cell death [30]. Glutamate is the primary excitatory neurotransmitter in the mammalian central nervous system (CNS), playing roles in synaptic plasticity, learning and memory and cognitive functions [18], [31]. During development, glutamate and glutamate receptor activation modulate cell proliferation, migration, neuronal differentiation and plasticity [32]. Aspartate, an excitatory neurotransmitter in the mammalian CNS (see [33], [34], [35]), functioning as a selective agonist of the ionotropic glutamate NMDA receptors [36], is enriched in the embryonic brain [37], [38]. The regional difference in aspartate level changes, its localization in the neuronal soma then in the axons once a distinct axonal layer has been established, the intense immunoreactivity within the cortical plate and subventricular zone of the early postnatal rat brain [39], support its involvement in neuronal differentiation and development. Early expression of NMDA and AMPA ionotropic glutamate receptors subunits accounts for their involvement in the establishment of synaptic contact and the refinement of neuronal circuits [32]. It is suggested that synaptogenesis triggers NMDA receptor clustering at nascent glutamatergic synapses, allowing NMDA receptors to regulate dendritic spine formation, maturation, and synaptic refinement and stabilization by controlling the pruning and strengthening of immature synapses during development [40]. In fact, dysfunction of glutamatergic synapses has been proposed as a pathogenic mechanism underlying neurodevelopmental disorders, including autism spectrum disorders, intellectual disability, Down syndrome, Rett syndrome and attention-deficit hyperactivity disorder [40], [41]. A reduction of aspartate content during embryonic phases, when critical neurodevelopmental processes, including generation of neuronal circuitry, are under the regulatory control of NMDA receptors, has been hypothesized to have potential clinical relevance, being possibly associated with vulnerability to develop psychotic-like symptoms [36]. Therefore, considering the importance of glutamate and aspartate signalling during brain development, in the present study we evaluated these two neurotransmitters.

We have previously reported that neurotransmitter release can be measured by high-performance liquid chromatography (HPLC) in rat cortical neurons, proving to be a very sensitive and accurate technology for the analysis of glutamate and aspartate levels [42], [43], [44]. In our recently published paper, we demonstrated that human iPSC-derived neuronal cultures are suitable to measure neurotransmitter release [45]. Therefore, in the present study, we applied this HPLC technology to measure the release of neurotransmitters in a previously well characterized hiPSC-derived NSC culture undergoing differentiation towards a mixed population of neurons and glia [2], [46], [47]. We assessed glutamate release in control cultures and upon exposure to DL-threo-β-Hydroxyaspartic acid (DL-TBOA, a non-transportable glutamate uptake inhibitor [48]), 4-aminopyridine (4-AP, which induces action potential-like membrane depolarization by blocking voltage-gated K^+^-channels, causing in vitro release of glutamate, [49], [50]), bafilomycin A1 (Bafilo, a specific and potent inhibitor of vacuolar-type H^+^-ATPase required for the exocytotic vesicle loading with glutamate [51], [52], [53]), or a combination of 4-AP and Bafilo.

Finally, we assessed whether repeated dose treatment (for 14 days) with some well-documented DNT compounds, i.e., Lead-II-Chloride (Lead), 2,2′4,4′-tetrabromodiphenyl ether (BDE47) and three different chemical mixtures had any impact on neurotransmitter release. Chemicals present in these mixtures were selected on the basis of several criteria, namely: (i) chemicals associated with cognitive/learning and memory impairment in children; (ii) chemicals acting through specific common key events in currently available DNT AOPs; (iii) chemicals representing different classes (i.e., pesticides, industrial chemicals, heavy metals, persistent organic pollutants, endocrine disruptors, and drugs); (iv) chemicals found in human samples (e.g., breast milk, cord blood, urine, hair, umbilical cord plasma, brain tissues, maternal blood, or children’s blood); and (v) chemicals working through similar and dissimilar mode of action (MoA), according to the EFSA definition [54]. We previously found that 14d treatment with these mixtures affected the generation of spontaneous electrical activity measured by multi-electrode array (MEA) technology in NSCs undergoing differentiation (from day 7 to day 21 of differentiation) [46].

The obtained data indicate that this human model is a suitable test system to measure the vesicular release of glutamate and aspartate both in control culture differentiated over several weeks (42 days) and upon exposure to neurotoxicants (single compounds or chemical mixtures). Importantly, analysis of neurotransmitter release turned out to be a sensitive and robust functional readout that should be considered for inclusion in the OECD DNT IVB of assays to support regulatory DNT testing.

## 2 Materials and methods

### 2.1 Differentiation of human induced pluripotent stem cell (hiPSC)-derived neural stem cells (NSCs) into mixed cultures of neurons and glia

The IMR90 cell line was originally developed at Coriell [55]. IMR90 fibroblasts were purchased and reprogrammed at I-Stem (Evry, France https://www.istem.eu/en/) by retroviral transduction of Oct4 and Sox2 using pMIG vectors (Addgene). IMR90-hiPSCs were kindly provided by Prof. Marc Peschanski, (I-Stem, France) to the JRC. Neural stem cells (NSCs) derived by the JRC from IMR90-hiPSCs were obtained by cutting hiPSC colonies into homogenous fragments; fragments were allowed to spontaneously generate embryoid bodies (EBs) under non-adherent conditions, which induced the formation of the three germ layers, as confirmed by qPCR analysis [56]. After 2 days, EBs were collected, seeded on laminin-coated dishes (10 µg/mL) (mouse protein, Merck-Sigma, cat. L2020) and cultured in the presence of neuroepithelial induction (NRI) medium [DMEM/F12, Thermo-Fisher, cat. 3133–038; Non-Essential Amino Acids, Thermo-Fisher cat. 11140–035; N2 Supplement, Thermo-Fisher, cat. 17502–048; Penicillin/Streptomycin (50 U/mL final concentration), Thermo-Fisher, cat. 15140–122, Heparin Grade I-A (2 µg/mL final concentration), Merck-Sigma, cat. H3149–100KU; bFGF (20 ng/mL final concentration, added before use), Thermo-Fisher, cat. 13256–029]. After about one week, IMR90-hiPSC neuroectodermal derivatives (called ‘rosettes’, characterized mainly by the presence of nestin+ cells (∼ 90%) and β-III-tubulin+ cells (∼ 5–10%)) were clearly visible. Rosettes were manually dissociated for further expansion of NSCs, as detailed in [2]. NSCs were subsequently expanded in T75 flasks coated with Matrigel Basement Membrane Matrix (Corning, cat. 354234, diluted 1:100 in cold DMEM/F12 medium), in the presence of neural induction (NI) medium [DMEM/F12 Thermo-Fisher, cat. 3133–038; Non-Essential Amino Acids, Thermo-Fisher, cat. 11140–035; Penicillin/Streptomycin (50 U/mL final concentration), Thermo-Fisher, cat. 15140–122; Heparin Grade I-A (2 µg/mL final concentration), Merck-Sigma, cat. H3149–100KU; B-27 Supplement (minus vitamin A), Thermo-Fisher, cat. 12587010; N2 supplement, Thermo-Fisher, cat. 17502–048; L-Glutamine (2 mM final concentration), Thermo-Fisher, cat. 25030–081, bFGF (10 ng/mL final concentration, added before use), Thermo-Fisher, cat. 13256–029, EGF (10 ng/mL final concentration, added before use), Thermo-Fisher, cat. PHG6045, and BDNF (2.5 ng/mL final concentration, added before use), Thermo-Fisher, cat. PHC7074].

NSCs were passaged using pre-warmed 0.05% Trypsin-EDTA (without phenol red, Thermo Fisher, cat. 15400054) for 2 min at 37 °C, and the enzymatic reaction was blocked with an equal volume of Defined Trypsin Inhibitor (Thermo Fisher, cat. R007–100). Cells were spun down at 1000 rpm for 4.5 min and viable cells counted using trypan blue and the Countess Automated Cell Counter (Thermo-Fisher, cat. C10227). After about five consecutive passages (performing each passage once per week when cells reached confluency), NSC cultures were characterized by the presence of nestin+ cells (∼ 55–62%), and morphologically immature neuronal cells stained for β-III-tubulin+ cells (∼ 22–25%), microtubule-associated protein-2 (MAP2)+ cells (∼ 15–18%), along with some immature glial fibrillary acidic protein (GFAP)+ cells (∼ 5–8%), assessed by immunocytochemistry and high content imaging as described in [1], [4]. NSCs were further differentiated into a mixed culture of neuronal and glial cells, in the presence of neuronal differentiation (ND) medium [Neurobasal Medium, cat. 21103049; N2 Supplements, B-27 Supplements, Penicillin/Streptomycin (50 U/mL), L-Glutamine (2 mM), laminin (mouse protein, 1 µg/mL), Merck-Sigma, cat. L2020, BDNF (2.5 ng/mL) and GDNF (1 ng/mL) (laminin, BDNF and GDNF were added before use) (all from ThermoFisher Scientific)], as previously described [1], [2].

When confluent, NSCs were passaged using 0.05% trypsin/EDTA and plated onto poly-D-lysine-coated 6-well plates (Nunc, cat. 152035) further coated with laminin (10 µg/mL) (mouse protein, Merck-Sigma, cat. L2020) (200,000 cells/well in 2 mL) for collection of supernatants and neurotransmitter release analysis. For analysis of electrical activity, cells were spot-plated onto sterile polyethylenimine (PEI) (from Merck-Sigma, cat. P3143) (0.1% solution in sodium-tetra-borate buffer) and laminin (mouse protein, 10 µg/mL) coated 24-well microelectrode array (MEA) plates (24-well glass MEA plate (24W300/30 G-288) V.232, from Multi-Channel System) in 100 µL volume, and after 20 min, 400 µL medium/well was added (50,000 cells/well in 0.5 mL). Further details about model characterization are provided in our previous studies [2], [47].

### 2.2 Repeated dose treatments with Lead-II-Chloride, BDE47 and selected chemical mixtures

After 7d differentiation on MEA 24-well plates, cultures were treated with individual chemicals and mixtures (Table 1).

**Table 1:** Chemicals used in the three mixtures.

Chemical name	Abbreviation	Purity	CAS	Supplier	Catalogue n.	Solvent	Concentration (µM)
Bisphenol A	BPA	≥ 99%	80–05–7	Merck-Sigma	239658	DMSO	12.74
Chlorpyrifos	CPF	analytical standard	2921–88–2	Merck-Sigma	45395	DMSO	21.01
Lead(II) chloride	Lead	98%	7758–95–4	Merck-Sigma	268690–5 G	DMSO	0.0073
2,2′4,4′-tetrabromodiphenyl ether	BDE47	≥ 97%	5436–43–1	Merck-Sigma	91834	DMSO	17.62
Ethanol	EtOH	≥ 99.8%	64–17–5	Merck-Sigma	51976	-	106,810
Methylmercury(II) chloride	Methyl-Hg	analytical standard	115–09–3	Merck-Sigma	33368	DMSO	0.05
PCB No 138	PCB138	analytical standard	35065–28–2	Merck-Sigma	35494	DMSO	0.0593
Valproic acid sodium salt	VA	98%	1069–66–5	Merck-Sigma	P4543	milliQ purified water	2.10
Vinclozolin	Vincl	analytical standard	50471–44–8	Merck-Sigma	45705	DMSO	0.42
2,3,7,8-tetrachlorodibenzo-p-dioxin	TCDD	Certified Reference Material	1746–01–6	Accustandard	D-404 N	DMSO	0.60

Mixtures were prepared as described in [46], and combined as follows:

• mixture combining five similar MoA chemicals (5-Sim): BPA, CPF, Lead, BDE47 and EtOH;
• mixture with five dissimilar MoA chemicals (5-Diss): Methyl-Hg, PCB138, VA, Vincl and TCDD;
• mixture containing all 10 chemicals (10-All).

The concentrations indicated in Table 1 correspond to low-toxic concentrations (‘LOEAC-syn’, i.e., the lowest concentration causing a statistically significant perturbation of synapse number), as described in [46]. These concentrations were originally established on the basis of resazurin test (CellTiter Blue); in particular, dose-response curves for cell viability were generated to identify, for each chemical, moderately cytotoxic (IC_20_, concentrations causing a 20% decrease of cell viability), and low cytotoxic (IC_5_, concentrations causing a 5% decrease of cell viability, and IC_20_ divided by 100, i.e., IC_20_/100) concentrations, compared with solvent control cultures (0.1% DMSO). In that study, individual chemicals at these three selected concentrations were assessed for their effects on synaptogenesis (measured by SYP+ and PSD95 + overlapping puncta quantification using high content imaging analysis). The lowest concentration causing a statistically significant perturbation of synapse number was considered for each chemical to create the aforementioned mixtures. For further details, please refer to [46].

Chemicals and media were refreshed twice/week. Electrical activity by MEA and viability were assessed after 14d treatment (i.e., 21d differentiation) for all chemicals (Supplementary Fig 3), while neurotransmitter release was assessed on 0d, 7d and 14d treatment with BDE47, Lead and the three mixtures (corresponding to 7d, 14d and 21d of cell differentiation).

### 2.3 Immunocytochemistry of differentiated neuronal/glial cultures

After 42d in differentiation cells were stained as previously described [4]. Briefly, cells were fixed with 4% formaldehyde for 10 min, washed twice with PBS 1X (w/o calcium and magnesium) and stored in PBS 1X at 4 °C prior to staining. Cells were incubated in PBS 1X containing 0.1% Triton-X-100% and 3.5% bovine serum albumin (BSA) (permeabilizing/blocking solution) for 15 min at room temperature and for additional 15 min in the presence of blocking solution (PBS 1X containing 3.5% BSA ). Then cells were incubated overnight (about 16 h) at 4 °C with primary anti-nestin (mouse, 1:200, Merck-Sigma, cat. MAB5326), anti-Ki67 (rabbit, 1:100, Abcam, cat. ab16667), anti-microtubule associated protein 2 (MAP2) (chicken, 1:3000, Abcam, cat. ab5392), anti-synaptophysin (SYP) (rabbit, 1:300, Abcam, cat. ab32127), anti-post-synaptic density protein 95 (PSD95) (mouse, 1:300, Abcam, cat. ab13552), anti-β-III-tubulin (mouse, 1:500, Abcam, cat. ab41489), anti-glial fibrillary acidic protein (GFAP) (chicken, 1:500, Thermo Fisher, cat. 14–9892–82), anti-vesicular glutamate transporter 1 (VGlut1) (rabbit, 1:300, Abcam, cat. ab72311), indicative of glutamatergic neurons, and anti-GABAergic marker glutamate decarboxylase 67 (GAD67) (mouse, 1:100, Thermo Fisher, cat. MA5–24909) antibodies, diluted in blocking solution. The day after, cells were washed twice with PBS 1X and further incubated for 1 h with Dy-Light-conjugated secondary antibodies (1:500, all from Abcam), and nuclei were counterstained with DAPI (1 µg/mL, Thermo Fisher), diluted in blocking solution. Wells containing stained cells were filled up with PBS 1X (w/o calcium and magnesium) and stored in the dark at 4 °C prior to analysis. Representative pictures were taken using 10x, 20x and 40x objectives integrated in the ArrayScan™ XTI high content imaging platform (Cellomics).

### 2.4 Flowcytometry analysis of NSCs undergoing differentiation towards neurons and glia

To quantify the overall percentages of nestin+ , β-III-tubulin+ , GFAP+ and CNPase (2′,3′-Cyclic-nucleotide 3'-phosphodiesterase)+ cells, NSCs (0d Diff) and cultures at 14, 28 and 42d differentiation were analysed by flow cytometry. NSCs and their differentiated culture derivatives were washed once with pre-warmed PBS 1x (without calcium and magnesium), and enzymatically dissociated with pre-warmed 0.05% Trypsin-EDTA (without phenol red, Thermo Fisher, cat. 15400054) (considering 1 mL/well in a 6-well plate) for 2–4 min at 37 °C. Cell aggregates were mechanically dissociated by pipetting them about five times with a p1000 tip to generate a homogenous single cell suspension. The enzymatic reaction was blocked with an equal volume of Defined Trypsin Inhibitor (Thermo Fisher, cat. R007–100), cells were centrifuged at 1000 rpm for 4.5 min, resuspended in PBS 1x and counted by trypan blue exclusion to evaluate cell viability. All subsequent centrifugation steps were done at 1800 rpm for 4.5 min. Cells were first fixed with 0.1% formaldehyde in PBS 1x for 10 min at room temperature keeping tubes on a gyratory shaker set at 50 rpm, washed twice with cold PBS 1x, permeabilized and blocked for 15 min in cold PBS/3.5% BSA/0.1% Triton X-100 (perm-blocking buffer), and stained for 30 min at room temperature on a gyratory shaker with primary anti-nestin (rabbit, 1:100, Merck-Sigma, MAB5326), anti-β-III-tubulin (mouse, 1:100, Abcam, cat. ab41489), anti-GFAP (rabbit, 1:400, Thermo Fisher, cat. PA5–16291), and anti-CNPase (mouse, 1:400, Thermo Fisher, cat. MA5–31374) antibodies, diluted in cold perm-blocking buffer, considering 1 × 10^6^ cells per tube. Cells were washed twice with cold PBS and counterstained for 30 min at room temperature on a gyratory shaker in the dark with either goat anti-rabbit or goat anti-mouse IgG H&L - DyLight® 488 conjugated secondary antibodies (1:200, Abcam) diluted in cold perm-blocking buffer. Cells were washed twice with cold PBS, resuspended in 1 mL of cold PBS/3.5% BSA and transferred into flow cytometry tubes for analysis. One tube with 1 × 10^6^ cells was left unstained, another one was incubated with secondary antibodies only; these were used as negative controls to set the gates. Flow cytometry was performed using a CyFlow™ Space Flow Cytometer, measuring 10,000 events per tube. Three biological replicates (i.e., cells collected after 3 passages on different days) were performed.

### 2.5 Analysis of endogenous excitatory neurotransmitter release

For analysis of neurotransmitter release, NSCs were plated onto poly-D-lysine-coated 6-well plates (Nunc, cat. 152035) further coated with laminin (10 µg/mL) (mouse protein, Merck-Sigma, cat. L2020) (200,000 cells/well in 2 mL). Cells were differentiated for a maximum of 42d and once/week supernatants were collected for neurotransmitter release analysis. Medium change was performed the day before supernatant collection. To assess evoked glutamate release, cultures at 21, 28, 35 and 42d of differentiation were stimulated once/week for 20 min with: DL-threo-β-Hydroxyaspartic acid (DL-TBOA, 10 µM, a glutamate uptake inhibitor), 4-aminopyridine (4-AP, 1000 µM, a blocker of the voltage-dependent potassium channels, causing in vitro release of glutamate), bafilomycin A1 (Bafilo, 2 nM, specific and potent inhibitor of vacuolar-type H^+^-ATPases), or a combination of both 4-AP + Bafilo (all from Merck, dissolved in DMSO), followed by complete medium change.

Moreover, NSCs differentiated for 7d on 24-well MEA plates (50,000 cells/well in 0.5 mL) were treated for 14d with Lead, BDE47 and the three mixtures tested in our previous study [46], and on the 14th day of treatment, cells were exposed for 20 min to 1000 µM 4-AP as detailed below. Three biological replicates (i.e., cells collected after 3 passages on different days) were performed, considering three to four internal replicates per condition.

The amount of endogenous glutamate and aspartate released in the collected samples was measured by high-performance liquid chromatography (HPLC), as previously described [57]. The analytical method involved automatic precolumn derivatization (Waters Alliance; Milford, MA) with o-phthalaldehyde, followed by separation on a C18 reverse-phase chromatography column (Chrompack International, 10 cm 4.6 mm, 3 mm) and fluorimetric detection. An homoserine solution was used as an internal standard. The detection limit was 100 fmol/mL. The amount of endogenous glutamate and aspartate released in the supernatant samples was expressed as pmol/well. When the effects of drugs were investigated, the endogenous glutamate or aspartate in the first sample (fraction 1, F1) collected before exposure (or stimulus) to drugs (i.e., DL-TBOA, Bafilo, 4-AP and 4-AP+Bafilo) was retained as the 100% control value for each well; endogenous glutamate in the supernatant sample (fraction 2, F2) in the presence (or absence) of drugs was evaluated as pmol/well and as the percentage variation with respect to the corresponding F1 value. Two-to-four wells were run in the absence of drugs (control wells). The endogenous glutamate overflow was measured for each well by subtracting the pmol/well in F1 from the pmol/well in F2 and calculating the % variation with respect to F1. The drug-evoked overflow was then calculated by subtracting the overflow in the control wells.

When we assessed whether repeated dose treatment with Lead, BDE47 and three different chemical mixtures had any impact on neurotransmitter release, 100 µL supernatants were collected and the endogenous glutamate was measured three times, i.e., before starting chemical treatments (T0), after 7 days (T7) and 14 days of treatment (T14), comparing effects induced by chemicals with solvent control (0.1% DMSO) at the respective time point. To measure the time-dependent effects of tested chemicals on endogenous glutamate, we calculated the ratios T7/T0 and T14/T0.

Additionally, the effects of chemicals on the 4AP-evoked glutamate release (4-AP at 1000 µM for 20 min), were assessed at the end of chemical treatment (on the 14th day of exposure), assessing glutamate levels prior and after 4-AP stimulation.

Samples were stored in 1.5 mL tubes at − 80 °C prior to shipping in dry ice for analysis of neurotransmitter release at the University of Genoa. Data were calculated as overflow for each condition. Three biological replicates (i.e., cells collected after 3 passages on different days) were performed, considering three to four internal replicates per condition.

### 2.6 Electrophysiological measurements using multi-well microelectrode array (MEA)

NSCs were passaged with trypsin/EDTA and plated onto sterile PEI (0.1%) and laminin (10 µg/mL) coated 24-well MEA plates (24-well glass MEA plate (24W300/30 G-288) V.232) (50,000 cells/well in 0.5 mL, each well containing 12 gold microelectrodes). After 7d differentiation, cultures were treated for 14 days with single chemicals (Table 1) and the three chemical mixtures tested in our previous study [46], refreshing medium and treatments twice/week (on day 10, 14 and 17 of differentiation). On the 14th day of treatment (i.e., 21d differentiation), plates were left to equilibrate for about 2–3 min prior to baseline measurement. Moreover, cells treated with Lead, BDE47 or mixtures were exposed to 1000 µM 4-AP, recording spontaneous electrical activity for 5 min before adding 4-AP (‘basal’) and 1 min after exposure to 4-AP. Electrical activity was recorded using the Multi-well MEA-System (Multi Channel Systems MCS GmbH), considering a Sampling Rate of 20000 Hz, a Low-Pass Filter Cutoff Frequency of 3500 Hz, and a High-Pass Filter Cutoff Frequency of 1 Hz. Spike rate (number of spike/sec) and burst count (considering a burst as a train of at least 4 spikes occurring within 50 millisec, with maximum interval to start burst of 50 ms, maximum interval to end burst of 50 ms, minimum interval between bursts of 100 ms) were analysed. Spike detection was based on an automatic threshold estimation considering the following parameters: 20 individual segments, baseline duration (duration of each segment) of 100 ms, a rising edge of 5 St. Dev. and a falling edge of − 5 St. Dev, timing (dead time) of 3000 µs, cutouts pre trigger 1000 µs and post trigger 2000 µs, estimated for all wells. Data analysis of spike rate and burst count was done using the "Multiwell-Analyzer" software, analysing the full recording and considering only active wells (i.e., wells characterized by at least 3 active channels, each active channel with minimum 10 spikes/min, and a minimum amplitude of 10 µV).

The average of spikes number and bursts number of selected active electrodes within each well were normalized to solvent Ctr (cells exposed to 0.1% DMSO for the same period of time as those exposed to chemicals), or to ‘basal’ (i.e., cells not yet exposed to 4-AP). Three independent biological replicates were done, considering four internal replicates per condition.

### 2.7 Analysis of cell viability with CellTiter-Blue®

After 7d of differentiation, cells were treated for 14 days with single chemicals at the nominal concentrations indicated in Table 1 and mixtures to determine possible cytotoxic effects. Cell viability was measured by incubating cells with CellTiter-Blue® Reagent (final 1:6 dilution in cell culture medium) at 37 °C and 5% CO_2_ for 3.5 h. After the incubation, 100 µL medium/reagent were transferred into new plates accounting also for wells containing blanc solution (medium with CellTiter Blue reagent), and fluorescence was measured at 530–560 nm-/590 nm (excitation/emission) in a multiwell fluorimetric reader (Tecan). After blanc subtraction, data were normalised to the mean of solvent control cells (0.1% DMSO).

### 2.8 Statistical analysis

**Fig. 2:**
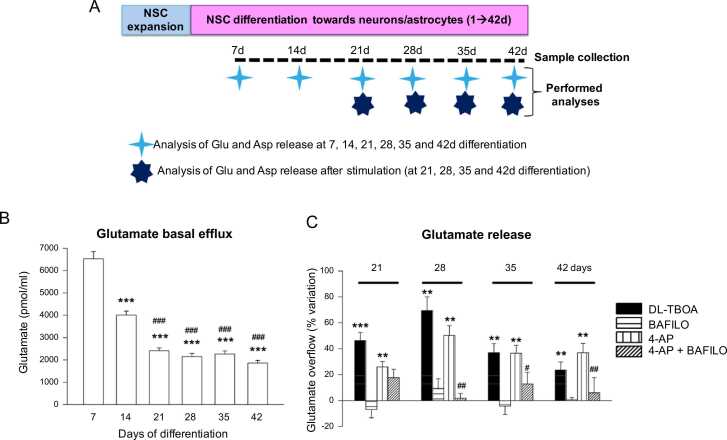
Analysis of glutamate release in control cultures undergoing differentiation and in cultures treated with DL-TBOA, Bafilo, 4-AP and 4-AP + Bafilo. (A) Schematic representation of experimental design: NSCs were expanded, passaged and differentiated up to 42 days, medium was refreshed twice/week. Supernatants were collected once/week for neurotransmitter release analysis (untreated cultures). Moreover, supernatants were collected at 21, 28, 35 and 42d differentiation before and after 20 min stimulation with DL-TBOA, Bafilo, 4-AP or both 4-AP+Bafilo. (B) The glutamate basal levels were measured by HPLC in cell supernatants collected at 7, 14, 21, 28, 35 and 42 days of differentiation. Bars represent the means ± S.E.M. in pmol/well of n = 3–5 different experiments. *** p < 0.001 vs glutamate level at 7d; ### p < 0.001 vs glutamate level at 14d (one way ANOVA and Tukey’s multiple comparisons test). (C) The evoked glutamate release overflow was measured in NSCs at 21–42 days of differentiation in response to DL-threo-β-Hydroxyaspartic acid (DL-TBOA, 10 µM, a glutamate uptake inhibitor; black bars), bafilomycin A1 (BAFILO, 2 nM, specific and potent inhibitor of vacuolar-type H^+^-ATPases; horizontal striped bars), 4-aminopyridine (4-AP, 1000 µM, a blocker of the voltage-dependent potassium channels; vertical striped bars), or a combination of both 4-AP + BAFILO (oblique striped bars). All the drugs were dissolved in DMSO (0.1% final DMSO concentration in medium) and at least two wells were considered as control. Data are expressed as mean ± S.E.M. from three different experiments, considering three to four internal replicates per condition. ** p < 0.01 and *** p < 0.001 vs control at the same day of differentiation; # p < 0.05 and ## p < 0.01 vs 4-AP (Kruskall-Wallis and Mann-Whitney test). For other experimental details see Materials and Methods.

**Fig. 3:**
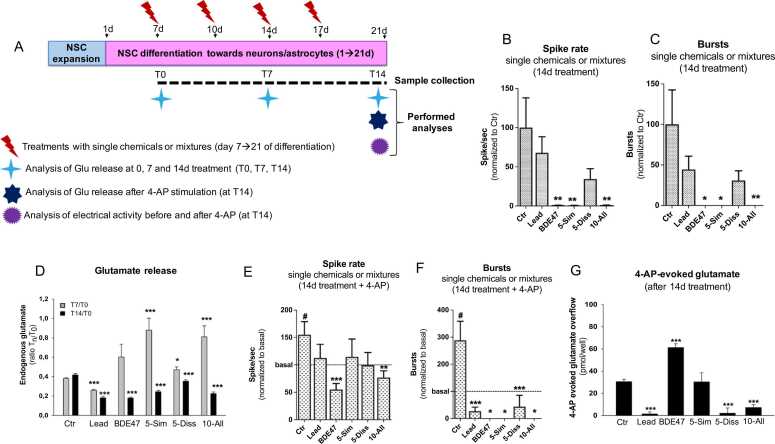
Analysis of neurotransmitter release and electrical activity in NSC cultures undergoing differentiation towards neurons/glia and treated with neurotoxicants for 14 days. (A) After 7d differentiation, NSCs were treated for 14 days with either Lead (0.0073 µM), BDE47 (17.62 µM) or three different chemical mixtures vs solvent control (Ctr, DMSO 0.1%) (for tested concentrations see Table 1). Chemicals and medium were refreshed twice/week (on day 10, 14 and 17 of differentiation). Supernatants were collected for analysis of glutamate release on day 0 (T0), day 7 (T7) and day 14 (T14) of treatment (corresponding to day 7, 14 and 21 of differentiation). Electrical activity was measured at T14, before and after stimulus with 4-AP (1000 µM). (B, C, E, F) Graphs show the effects of tested chemicals and mixtures after 14d treatment on spike rate (B) and bursts (C), and the same treatment conditions before (‘basal’) and after 1 min stimulation with 4-AP (E, F). For B and C, one-way ANOVA (with Dunnett post-test) was used, comparing each data set with solvent control (Ctr) (* p < 0.05, ** p < 0.01). For E and F, paired t test (two tailed) was used to compare the basal value (before 4-AP stimulation) vs 4AP-evoked effect in Ctr culture at T14 (# p < 0.05). Two-way ANOVA (with Dunnett post-test) was used to compare 4AP-evoked effect for each chemical treatment vs 4AP-evoked effect in control (Ctr) (* p < 0.05, ** p < 0.01, *** p < 0.001). Data were normalized to solvent Ctr (B, C), or to the basal value before 4-AP stimulation (at T14) (E, F) and are expressed as mean ± S.E.M. of n = 3 different experiments, considering four internal replicates per condition. (D) Graph shows the effects of tested chemicals and mixtures after 7d and 14d treatment on basal glutamate release. The glutamate levels were measured at T0, T7 and T14 in solvent control (Ctr) or chemical exposure conditions, and the ratios T7/T0 (grey bars) and T14/T0 (black bars) were calculated. Data are expressed as mean ± S.E.M. of n = 3 different experiments, considering three to four internal replicates per condition. * p < 0.05, *** p < 0.001 by Mann-Whitney test, comparing the ratios T7/T0 and T14/T0 obtained in control condition vs the same ratios calculated in the presence of chemicals at the same time. (G) Graph shows 4-AP ability to evoke endogenous glutamate assessed after 14d treatments (the overflow is shown). Data are expressed as mean (pmol/well) ± S.E.M. of n = 3 different experiments, considering three to four internal replicates per condition. *** p < 0.001 by Kruskall-Wallis and Mann-Whitney test comparing the glutamate efflux evoked by 4-AP in the presence of chemicals and in solvent control (Ctr). For other experimental details see Materials and Methods.

Statistical significance of neurotransmitter release data was assessed by one-way ANOVA and Tukey’s Multiple Comparisons Test, comparing the level of glutamate and aspartate at 0–42d of differentiation (Fig. 2B and Supplementary Fig. 2A), by the non-parametric Kruskall-Wallis analysis and Mann-Whitney test comparing the glutamate value measured in the presence of drugs and in control condition or comparing the glutamate released in the presence of 4-AP + Bafilo vs 4-AP alone (Fig. 2C). Statistical significance of neurotransmitter release data was assessed by Mann-Whitney test, comparing the ratios T7/T0 and T14/T0 obtained in solvent control (Ctr, 0.1% DMSO) condition vs the same ratios calculated in the presence of chemicals at the same time (Fig. 3D). To assess the ability of 4-AP to evoke glutamate release after chemical treatments, statistical significance of neurotransmitter release data was assessed by Kruskall-Wallis and Mann-Whitney test, comparing the glutamate value measured in the presence of chemicals and in control condition (Ctr, solvent control cell, DMSO 0.1%; Fig. 3G). Statistical significance of MEA analysis was assessed by one-way ANOVA with Dunnett's Multiple Comparison Test, comparing chemical treatments’ effect vs solvent control (Ctr; Fig. 3B, C). Paired t test (two tailed) was also used to compare ‘basal’ vs 4-AP-evoked effect only in solvent ‘Ctr’ culture (Fig. 3E, F). Two-way ANOVA (with Dunnett post-test) was used to compare 4-AP-evoked effect for each chemical treatment vs solvent ‘Ctr’ (Fig. 3E, F). Statistical significance of flowcytometry analysis was assessed by one-way ANOVA with Dunnett's Multiple Comparison Test, comparing cell populations percentages at different time points vs NSCs (0d differentiation) (Supplementary Fig. 1C). GraphPad Prism 9 software was used to compile and analyse data, which represent the average of at least 3 biological replicates ± standard error mean (S.E.M.). For all graphs, an asterisk (*) or a hash (#) over a data point indicates a significant difference between different conditions as indicated in each figure legend (* and # p < 0.05; ** and ## p < 0.01; *** and ### p < 0.001).

## 3 Results

Human iPSC-derived NSCs were differentiated for 42d towards a mixed culture of neuronal/glial cells as described in [2] (Supplementary Fig 1A). The proportion of Nestin+ and Ki67 +cells, indicative of proliferating NSCs, decreased significantly over differentiation (Supplementary Fig. 1B, C); in particular, after 42 days, the proportion of remaining nestin+ cells was about 10–12% of total cell number (Supplementary Fig. 1C). Accordingly, nestin and Ki67 levels assessed by IC and high content imaging analysis have been reported in our previous study [4] (See Fig. 1 in that paper), which showed a time-dependent downregulation during differentiation.

On the other hand, the proportions of neurons and astrocytes significantly increased over time: at 42d, the percentage of β-III-tubulin+ neurons was about 60–70%, while GFAP+ astrocytes represented about 40–45% of total cell number (Supplementary Fig. 1B, C). The expression of CNPase, indicative of oligodendrocytes, was very modest (about 4% of CNPase+ cells after 42d of differentiation) and the percentage of CNPase+ cells did not significantly change over time (Supplementary Fig. 1C), as previously reported [47].

Moreover, differentiated cultures showed expression of microtubule-associated protein 2 (MAP2), characteristic of dendrites [58], and the synaptogenesis-related proteins synaptophysin (SYP, pre-synaptic) and post-synaptic density 95 (PSD95), known to be expressed in excitatory neurons (Supplementary Fig. 1B). Noteworthy, at 42d of differentiation, the vast majority of neurons were positive for the vesicular glutamate transporter 1 (VGlut1) (about 80–85% of β-III-tubulin+ neurons), which is expressed by glutamatergic neurons [59], with a low proportion of cells positive for GAD67 (about 10% of β-III-tubulin+ neurons) (Supplementary Fig. 1D, E), one of the enzymes controlling the synthesis of the inhibitory neurotransmitter GABA [60]. The presence of functional excitatory glutamatergic neurons was also confirmed by cell culture responsiveness to CNQX stimulation, an AMPA and kainate receptor antagonist, as described in [47]. In addition, in our previous study [47] we reported that this NSC culture undergoing differentiation for up to 42 days, underwent time-dependent upregulation of genes associated with glutamatergic, GABAergic, dopaminergic and cholinergic neuronal cells. Further characterization of this cellular model is described in [1], [2], [3], [46].

For analysis of neurotransmitters’ release (glutamate and aspartate), NSCs were differentiated for up to 42d; starting from 7d differentiation, supernatants were collected once a week for analysis of basal neurotransmitter release and upon addition of DL-TBOA, Bafilo, 4-AP and 4-AP+Bafilo at selected time points (Fig. 2A), as described in the following section.

### 3.1 Analysis of neurotransmitter release in control cultures and in response to DL-TBOA, Bafilo, 4-AP and 4-AP + Bafilo stimulation

Analysis of neurotransmitter release revealed that NSCs undergoing neuronal/glial differentiation were able to release glutamate. In these cultures, basal efflux of glutamate decreased during differentiation in the first two weeks and the extracellular glutamate reached steady state levels starting from 21d of differentiation (Fig. 2B).

We then tested the effects of pharmacological treatments in order to investigate the mechanisms controlling the maintenance of extracellular glutamate levels in mature neuron-astrocyte networks, namely glutamate release (especially the depolarization-evoked vesicular release) and its clearance (i.e., glutamate uptake through the excitatory amino acid transporters, EAATs), in cells differentiated for 21d, 28d, 35d and 42d. We found that the EAAT inhibitor DL-TBOA increased the extracellular glutamate levels at all the times considered (Fig. 2C), suggesting that EAATs are functioning and are involved in glutamate clearance. Notably, 4-AP was able to evoke glutamate overflow at all the times considered (Fig. 2C). Furthermore, Bafilo, an inhibitor of the v-ATPase required for the exocytotic vesicle loading with glutamate, per se ineffective on the glutamate efflux, inhibited the 4-AP-evoked glutamate overflow at 28d, 35d and 42d, indicating vesicular exocytotic release of glutamate (Fig. 2C). Supplementary Fig. 2 shows the ability of NSCs undergoing neuronal/glial differentiation to release aspartate, particularly upon stimulation with 4-AP, and the inhibitory effect of Bafilo on 4-AP-evoked aspartate overflow at 21–42 days of differentiation (Supplementary Fig. 2A, B).

### 3.2 Impact of Lead, BDE47 and selected chemical mixtures on electrical activity and glutamate release

We previously showed that repeated exposure for 14 days of NSCs undergoing neuronal/glial differentiation to selected single chemicals at low-cytotoxic concentrations (see Table 1 and [46]) and to mixtures of the same chemicals, affected synapse formation, as well as the generation of spontaneous electrical activity [46]. In our previous study, the resulting mixtures were composed as follows: a mixture combining five similar MoA chemicals (5-Sim: BPA, CPF, Lead, BDE47 and EtOH), a mixture with five dissimilar MoA chemicals (5-Diss: Methyl-Hg, PCB138, VA, Vincl and TCDD), and a mixture containing all ten chemicals (10-All). The three chemical mixtures were created considering the MoA of individual compounds, which are all known to be present in biological samples of children and their mothers (e.g., breast milk, maternal and child blood, cord blood), to be associated with neurocognitive impairments in children, and to affect synaptogenesis (see references reported in Tables S1 and S2 of [1], and Table S1 of [46]. However, while similar MoA chemicals have been shown to alter BDNF signalling, dissimilar MoA chemicals act through different MoAs (for more details on chemical selection, refer to [46]).

The chemical concentrations reported in Table 1 resulted as the Lowest Observable Adverse Effect Concentration (LOAEC) suitable to perturb synaptogenesis (measured by SYP+ and PSD95 + overlapping puncta quantification) (i.e., the lowest concentration causing a statistically significant perturbation of synapse number), as described in [46]. These concentrations were selected in this study for further analysis of spontaneous electrical activity and neurotransmitters’ release.

We found that both the mixture containing five chemicals with similar MoA (5-Sim) and the one with all ten chemicals (10-All), caused a remarkable decrease of electrical activity after 14d treatment compared to solvent control treated cells (0.1% DMSO) (see normalized data in Fig. 3A-C for selected chemicals (i.e., BDE47, Lead and mixtures), and raw data for all individual chemicals and mixtures in Supplementary Fig. 3), in line with our previous data [46]. The main trigger of such effects was BDE47 when tested at a concentration of 17.6 µM (corresponding to an IC_5_ on the basis of dose-response curve for cell viability assessed by CellTiter Blue (resazurin) test, as described in [46]). Indeed, BDE47 at this concentration caused a strong decrease of both spike rate (Fig. 3B), and the number of bursts (Fig. 3 C) (Supplementary Fig. 3B, C). Moreover, only a tendency towards a decrease of these electrical parameters (although not significant) could be observed in cells treated with Lead administered alone (0.0073 µM, very low-cytotoxic, on the basis of dose-response curve for cell viability assessed by CellTiter Blue (resazurin) test in [1]), and the mixture containing five chemicals with dissimilar MoA (5-Diss) (Fig. 3B, C). As shown in our previous study, other compounds present in the three mixtures at these tested concentrations did not cause significant changes of electrical activity [46] (Supplementary Fig. 3B, C).

Noteworthy, after 7d of exposure, a tendency toward an increase of extracellular glutamate was found for BDE47, while tested chemical mixtures (5-Sim, 5-Diss and 10-All) significantly increased the levels of extracellular glutamate (Fig. 3D, grey bars). On the contrary, a significant decrease of glutamate level was found in cultures treated for 7d with Lead compared to solvent Ctr (Fig. 3D, grey bars). After a prolonged period of time (14d), cells exposed to Lead, BDE47, 5-Sim, 5-Diss and 10-All showed a reduced release of basal glutamate (Fig. 3D, black bars), which could explain the general decrease of spontaneous electrical activity observed after 14d treatment with these compounds (Fig. 3B, C).

Furthermore, we sought to investigate the specific effects elicited by 4-AP (1000 µM) on electrical activity in cultures exposed to BDE47, Lead and mixtures for 14d. MEA data showed that 1 min stimulation with 4-AP caused an increase of spike rate (by about 50% vs ‘basal’) and bursts (about 2.9-fold increase vs ‘basal’) in solvent control cultures (0.1% DMSO) (Fig. 3E, F). On the contrary, in cells that were treated with either BDE47 alone or the 10-All mixture, a reduced spike rate in response to 4-AP (about 20% lower than ‘basal’) was observed (Fig. 3E). The number of bursts were already strongly decreased after 14d treatment with BDE47, 5-Sim and 10-All mixtures (Fig. 3C), and upon 4-AP stimulation, all treatment conditions showed zero bursts compared to solvent control culture (Fig. 3F).

Regarding possible cytotoxic effects of tested chemical mixtures and individual chemicals, we previously showed by CellTiter-Blue (resazurin) test that while individual chemicals were generally not-cytotoxic or very low-cytotoxic, decrease of cell viability (or cytotoxicity) could be observed upon 14d treatment with 5-Sim and 10-All mixtures (between 55% and 65% decrease compared to solvent control at the respective time point) (Supplementary Fig. 3A), as previously reported [46]. Therefore, the decrease of electrical activity observed in cultures treated with these two mixtures may (also) be associated with cytotoxicity and/or cell viability decrease.

On the other hand, the remarkable decrease of electrical activity induced by BDE47, the tendency towards a reduction of electrical activity elicited by Lead and 5-Diss mixture (although not significant, see Fig. 3B,C), and observed also upon exposure to 4-AP (Fig. 3E,F), may be likely associated with specific effects on the glutamate system, which could imply e.g., an inhibition of glutamate release caused by chemical treatment(s), or induction of excitotoxicity associated with over-activation of glutamate receptors occurring e.g., as a result of increased release or decreased uptake of glutamate [61]. To investigate the mechanisms underlying these effects, we measured the level of glutamate in the extracellular medium in all these treatment conditions, before and after 20 min stimulation with 4-AP. We found that individual Lead exposure and 5-Diss mixture almost abolished the ability of 4-AP to evoke the release of glutamate (Fig. 3G). A similar effect was observed also with 10-All mixture (Fig. 3G), although, as commented above, cytotoxicity may also play a role in the induction of these effects. On the other hand, 5-Sim mixture did not affect 4-AP-evoked glutamate overflow; however, cytotoxicity may also be involved in this effect. This may suggest inhibition of glutamate release under these treatment conditions, especially for Lead and 5-Diss mixture. On the other hand, BDE47 was found to increase the 4-AP-evoked glutamate efflux (Fig. 3G), possibly suggesting induction of excitotoxicity.

## 4 Discussion

### 4.1 Human NSCs differentiated towards neuronal/glial cultures release glutamate: glutamate clearance and vesicular release concur to maintain extracellular glutamate level

Here we show that human NSCs differentiated towards a mixed population of neuronal/glial cells have the ability to release glutamate (and aspartate), reaching steady state of extracellular levels starting from the 21st day of differentiation. This suggests maturation and dynamic equilibrium of the mechanisms regulating extracellular glutamate levels, i.e., the mechanisms involved in glutamate release, as well as those controlling glutamate clearance. Prior to synapse formation, when the density of synapses is relatively low and networks are still maturing, an early paracrine glutamate communication is present [62]. From the 21st day of differentiation, the ability of DL-TBOA to increase extracellular glutamate levels suggests that EAATs are functional in this cellular model and control glutamate clearance. Additionally, cells were also able to respond to depolarization by releasing both glutamate and aspartate. Inhibition of the 4-AP-evoked glutamate or aspartate overflow by Bafilo, an inhibitor of the v-ATPase required for the exocytotic vesicle loading with the excitatory neurotransmitters, indicates the ability of 4-AP to evoke vesicular exocytotic release of glutamate and aspartate. Although we are aware of some skepticism about aspartate being a neurotransmitter [63], D-aspartate was reported to be taken up into the nerve terminals and concentrated in synaptic vesicles [64], [65], [66]. Aspartate is subjected to exocytotic release from cultured neurons, isolated nerve terminals, and brain tissue [33], [34], providing essential support to the hypothesis that aspartate can be considered *de facto* as an endogenous neurotransmitter.

To our knowledge, the ability of human NSCs undergoing differentiation towards neuronal/glial cells to release endogenous excitatory neurotransmitters in a vesicular manner has not been described yet. In line with the present work, we have previously reported a Ca^2+^-dependent release of the glutamate analogue 3 H-D-aspartate evoked by depolarization in hiPSC-derived neurons, indicating the maturation of the vesicle exocytotic machinery [45].

Neuronal cells described in this study are mainly characterized by the presence of VGlut1, indicating their glutamatergic nature, and express the presynaptic marker SYP and the post-synaptic marker PSD95, typical of glutamatergic synapses, indicative of development of glutamatergic synaptic structures. The presynaptic compartment appeared to be functional, as indicated by the development of vesicular release. The presence of functional excitatory glutamatergic neurons and glutamatergic activity was also confirmed by cellular responsiveness to blockade of glutamatergic AMPA and kainate receptors (by CNQX), as described in our previous study [47]. Moreover, the observed blockage of EAATs indicates that the machinery controlling glutamate clearance from the synapses is functional. This could be attributed to the presence of GFAP+ astrocytes, which play an important role in the reuptake of glutamate in the proximity of the synaptic cleft [67].

Altogether, these findings demonstrate that hiPSC-derived NSCs can differentiate into a functional network of mature mixed neuronal and glial cells as confirmed by synchronized spontaneous activity recorded using MEA reported in our previous study [47], and the glutamatergic transmission investigated in the present study. This is of relevance considering that impairment of glutamatergic transmission represents a key event in adverse outcome pathways (AOPs) relevant to neurotoxicity [68], [69]. We showed that the impact of neurotoxicants (single chemicals or mixtures) on both the release of glutamate and functioning of EAATs, can be measured in this human model using the described assay, enabling more reliable screening of chemicals that could trigger this MoA.

### 4.2 Analysis of neurotransmitter release and electrical activity as sensitive functional endpoints for DNT testing

Although the neuronal network electrical activity may depend on ongoing activation of several neurotransmitter systems, the ability of 4-AP depolarization to evoke an increase of both firing rate and spike trains (bursts), paralleled by an increase in glutamate release, confirms the main role of glutamate in regulating network activity. This also suggests that studying neuronal network function by measuring both electrical activity and release of neurotransmitters, represents a reliable approach to evaluate this functional endpoint, and should be implemented in the current IVB of tests for regulatory DNT testing. As shown in this study, simultaneous analysis of neurotransmitter release and electrical activity in cultures of mixed neuronal and glial cells treated with neurotoxicants, confirms the ability of some neurotoxicants to affect both these functional parameters, permitting better mechanistic understanding of the observed effects.

Our data showed that after 7-day exposure to selected chemicals, a tendency toward an increase of extracellular glutamate was found for BDE47, while a significant increase of extracellular glutamate was found in cultures treated with tested mixtures (5-Sim, 5-Diss and 10-All). On the contrary, a decrease of glutamate was found in cultures treated with Lead. After a longer period of time (14 days), a general reduction of glutamate extracellular levels was observed at all tested conditions, which may be possibly due to a compromised neuron ability to synthesize or release glutamate. This may be related to the observed decrease of spontaneous electrical activity after 14d treatment (significant for BDE47, the 5-Sim and 10-All mixtures, both containing BDE47). In fact, the depolarization-evoked electrical activity was greatly reduced after exposure to these chemicals. It should also be considered that the observed increase of glutamate levels possibly leading to excitotoxicity (especially occurring after 7d exposure to 5-Sim and 10-All), may be responsible for the induction of cytotoxicity observed after 14d exposure to the same mixtures.

In parallel, glutamate release triggered by the depolarizing stimulus (with 4-AP) was decreased in cells treated with Lead and 5-Diss mixture, consistent with the observed network electrical activity inhibition trend. Conversely, an increase in glutamate release in response to depolarization was found in cultures exposed to BDE47, which may be due to inhibition of glutamate re-uptake induced by BDE47 [70]. The lack of effect of 5-Sim mixture (containing both Lead and BDE47), may also be a result of the opposing effects of these two compounds on the evoked glutamate release.

With regards to BDE47, along with its hydroxylated metabolites, it has been shown to decrease the maturation and functionality of embryonic rat cortical neurons, dysregulating the expression of neuronal activity-related genes [71]. Inhibition of MEK-ERK signalling associated with electrical activity impairment, decrease of pre-synaptic functions and axon guidance have also been linked to BDE47 effects [72], supporting our observations on spontaneous electrical activity inhibition.

Noteworthy, some of the concentrations of chemicals tested in our previous studies [46], as well as in the present study, correlate with concentrations found in human samples (mainly cord blood or maternal or fetal blood). For instance, in cord and maternal blood, the concentrations of Lead have been described in the range of ∼ 0.004–0.13 μM (see Table 1 in [1]), calculated on the basis of studies, such as [73], [74], [75].

On the other hand, some of the tested chemical concentrations are higher than levels found in epidemiological studies. For instance, concentration of BDE47 tested in the present study (17.6 μM) is higher than levels found in biological samples (calculated in the range of 6.1 × 10^−5^ - 0.038 μM, on the basis of concentrations found in human samples), as described in [46], and cited human studies (e.g., [76], [77], [78]). Nevertheless, the nominal concentrations of chemicals present in the mixtures and considered for the analysis of the selected DNT endpoints are in the range of concentrations described in previously published in vitro studies (e.g., [79], [80], [81], [82], [83]).

### 4.3 Study of the neurotransmitter release may contribute to better mechanistic understanding of developmental neurotoxicity

The obtained results contribute to better understanding the neurotoxicity mechanisms of tested chemicals, in particular BDE47 and Lead. While an impairment of glutamatergic transmission can be well-related to a reduction of network electrical activity, which we found to be mainly sustained by glutamatergic transmission in this cell model [47], a dramatic activity reduction (or blockage) can depend on neuronal cell loss, occurring e.g., as a consequence of increased glutamate extracellular levels, activation of ionotropic glutamate receptors, and excitotoxicity. In particular, excitotoxicity is a common neurotoxic pathway in acute neuronal damage (e.g., during ischemia) and chronic neurodegenerative conditions [84], and is identified as one of the key events involved in neurotoxicity mechanism described in AOPs [68]. Excitotoxicity eliciting neuronal cell death also contributes to learning and memory impairment in adults, as described in the AOP 48 [85].

Consistent with our findings on evoked glutamate release, BDE47 was suggested to cause an increase of extracellular glutamate levels in mouse cerebellar neurons, which in turn activates ionotropic glutamate receptors leading to increased calcium levels, oxidative stress, and cell death [86]. In NPCs, this neurotoxicant inhibited axon outgrowth and neuronal connectivity [87], and induced upregulation of stress response and of glutamate receptors’ transcription [88].

On the contrary, a cascade of key events triggered by inhibition of glutamate release upon exposure to Lead has been described in AOP 13. Lead is known to act as an antagonist of NMDA receptor, causing its blockage and finally leading to impairment of learning and memory in children, which was identified as an adverse outcome in AOP 13 [89]. One of the identified key events in this AOP is reduced intracellular calcium levels, affecting cellular functions [90] and reduced glutamate release in vivo [91], [92], [93], in line with our findings. Moreover, Lead at nanomolar concentration has been shown to block glutamate (and GABA) evoked release in cultured hippocampal neurons in vitro [94], as well as in brain slices [93]. Consistent with our results, 5d exposure to Lead has been reported to dysregulate presynaptic protein expression and decrease vesicular glutamate release [95], [96]. Further molecular mechanisms underlying perturbation of glutamate release or clearance in control cultures and upon exposure to single chemicals and chemical mixtures (e.g., by analysis of glutamate receptor-related and EAAT-related gene expression) have not been explored in the present study and may warrant further investigation.

In conclusion, measuring extracellular glutamate levels may help clarify the mechanisms underlying neurotoxicant-induced neuronal damage and neuronal loss, contributing to better mechanistic understanding of DNT effects.

## 5 Conclusions

To sum up, human NSCs undergoing differentiation towards a mixed culture of neuronal/glial cells have the ability to release glutamate; glutamate clearance and vesicular release both concur to maintain the extracellular glutamate levels. Analysis of neurotransmitter release along with electrical activity represent complementary functional endpoints for reliable in vitro DNT testing. In particular, neurotransmitter release analysis applied to human NSC-derived neuronal/glial cell models represents a sensitive readout that may contribute to mechanistic knowledge and should be included in the envisioned battery of DNT testing in vitro [13]. Considering readiness as a criterion for DNT assay set-up [97], [98], the described HPLC assay can be qualified as medium-to-high throughput, which would make this assay suitable for its inclusion in the battery.

## Declaration of Competing Interest

The authors declare that they have no conflict of interests.
