# Supplementary material for: Assessment of neurotransmitter release in human iPSC-derived neuronal/glial cells: a missing in vitro assay for regulatory developmental neurotoxicity testing
Source: Reprod Toxicol. 2023 Apr;117:108358. doi: 10.1016/j.reprotox.2023.108358 (PMC10112275; doi:10.1016/j.reprotox.2023.108358)
Supplement: Supplementary file 1 — Supplementary material [file mmc1.docx]

**Supplementary Figures**

**Supplementary Figure 1. Human iPSC-derived NSC differentiation towards a mixed neuronal/glial culture.** (A) Representative phase contrast images of undifferentiated human NSCs (after 5d in expansion) and cells differentiated for 42d towards a mixed culture of neuronal and glial cells. (B) Representative fluorescent images of human NSCs differentiated for 42d; cells were stained for Nestin (green)/Ki67 (red), β-III-tubulin (red)/GFAP (green), MAP2 (white)/SYP (green)/PSD95 (red), and VGlut1 (green)/GAD67 (red) along with DAPI (using a 10x objective, with insets images taken using a 20x objective). (C) Bar chart showing the proportion of nestin+, β-III-tubulin+, GFAP+ and CNPase+ cell populations in NSCs (0d differentiation, light blue bars), and in cultures differentiated for 14d, 28d, and 42d. One-way ANOVA (with Dunnett post-test) was used to compare 14d, 28d and 42d differentiation with 0d differentiation (i.e., undifferentiated NSCs) (* p < 0.05, *** p < 0.001). Data are shown as mean ± S.E.M. of 3 biological replicates. (D) Quantification of VGlut1+ and GAD67+ neuron percentages on total β-III-tubulin+ cells (mean ± S.E.M. of 3 biological replicates, considering manually counting at least 5 pictures for each passage). (E) Representative fluorescent images of differentiated (42d) cells stained for DAPI, β-III-tubulin (red) and VGlut1 (green) showing individual staining (pictures were taken using a 40x objective).

**Supplementary Figure 2. Analysis of aspartate release in control cultures undergoing differentiation and in cultures added with Bafilo, 4-AP and 4-AP+Bafilo.** (A) The aspartate basal levels in hiPSC-derived NSCs were measured by HPLC in samples collected at 7, 14, 21, 28, 35 and 42 days of differentiation. Bars represent the means ± S.E.M. of pmol/well related to n= 3-5 different experiments. ***p < 0.001 or ** p < 0.01 vs aspartate level at 7d; ### p < 0.001 or ## p < 0.01 vs aspartate level at 14d (one-way ANOVA and Tukey’s multiple comparisons test). (B) The evoked aspartate release overflow was measured from hiPSC-derived NSCs at 21-42 days of differentiation in response to DL-threo-β-Hydroxyaspartic acid (DL-TBOA, 10 µM, a glutamate uptake inhibitor; black bars), bafilomycin A1 (Bafilo, 2 nM, specific and potent inhibitor of vacuolar-type H^+^-ATPases; horizontal striped bars), 4-aminopyridine (4-AP, 1000 µM, a blocker of the voltage-dependent potassium channels; vertical striped bars), or a combination of both 4-AP + Bafilo (oblique striped bars). All the drugs were dissolved in DMSO (0.1%) and at least two wells were considered as control. Data are expressed as mean ± S.E.M. from three different experiments. **p<0.01 and ***p<0.0001 vs control at the same day of differentiation; # p< 0.05 and ## p< 0.01 vs 4-AP (Kruskall-Wallis and Mann-Whitney test). For other experimental details see Materials and Methods.


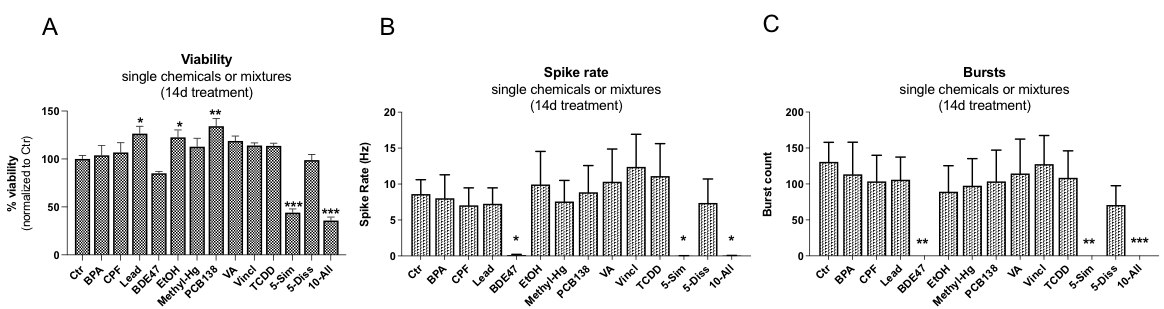


**Supplementary Figure 3. Cytotoxicity and raw data of MEA analysis. (A)** Graph showing cytotoxicity (or viability) of NSCs exposed during differentiation to single chemicals and mixtures for 14 days (from 7 to 21d of differentiation). CellTiter blue analysis was performed on the last day of treatment (data shown in this graph have been published in (Reprod Toxicol. 2021 Oct;105:101-119., see Figure 3D, red curve). (B, C) Graphs showing raw data of MEA analysis of the effects of individual chemicals and mixtures after 14d treatment (spike rate (A) and bursts (B)). For all graphs, one-way ANOVA (with Dunnett post-test) was used, comparing each data set with solvent control (Ctr, 0.1% DMSO) (* p < 0.05, ** p < 0.01, *** p < 0.001).
